# Mechanism and physiological significance of autoregulation of the *Escherichia coli hfq* gene

**DOI:** 10.1261/rna.068106.118

**Published:** 2019-02

**Authors:** Teppei Morita, Hiroji Aiba

**Affiliations:** Faculty of Pharmaceutical Sciences, Suzuka University of Medical Sciences, Suzuka, Mie, 513-8670, Japan

**Keywords:** Hfq, autogenous control, translation, RNA-binding surfaces, 5′-UTR

## Abstract

The RNA chaperone Hfq plays a critical role in sRNA-mediated gene regulation in enteric bacteria. The major role of Hfq is to stimulate base-pairing between sRNAs and target mRNAs by binding both RNAs through three RNA-binding surfaces. To understand the post-transcriptional network exerted by Hfq and its associated sRNAs, it is important to know how the cellular concentration of Hfq is regulated. While an early study showed that *hfq* translation is repressed by Hfq, the detailed mechanism and biological significance of the *hfq* autoregulation remain to be studied. Here, we show that the synthesis of Hfq is strictly autoregulated to maintain the cellular concentration of Hfq within a limited range even when the *hfq* mRNA is overexpressed from a plasmid-borne *hfq* gene. Mutational and biochemical studies demonstrate that Hfq represses its own translation primarily by binding to the *hfq* mRNA through the distal face. The growth of cells harboring the *hfq* plasmid is markedly inhibited due to an increased Hfq level when the distal face of Hfq is mutated or the 5′-UTR of *hfq* is mutated. A mutation in the rim suppresses the growth inhibition caused by the distal face mutation, suggesting that the interaction of Hfq with undefined RNAs through the rim is responsible for the growth inhibition by the increased Hfq level. In addition, the data suggest that the *hfq* autoregulation operates not only in cells harboring a multicopy *hfq* gene but also in the wild-type cells.

## INTRODUCTION

The RNA-binding protein Hfq was originally identified as a host factor required for the replication of bacteriophage Qβ in *Esherichia coli* (Franze de Fernandez et al. 1968). It is now recognized that the protein acts as a pleiotropic regulator to modulate the stability and the translation of a number of RNAs in bacteria. In particular, Hfq plays the key role in the post-transcriptional control of gene expression, acting as an RNA chaperone, along with its associated regulatory small RNAs (sRNA) in gram-negative bacteria (Vogel and Luisi 2011; Sobrero and Valverde 2012; Updegrove et al. 2016; Kavita et al. 2018). The Hfq-dependent sRNAs are induced in response to specific physiological/stress conditions and stabilized by Hfq. In addition, modulation of transcription termination also contributes to an efficient generation of functional sRNAs (Morita et al. 2015, 2017). The major role of Hfq in sRNA-mediated gene regulation is to facilitate base-pairing between sRNAs and target mRNAs by binding both RNAs although additional layers of RNA-based regulation by Hfq and sRNAs continue to be found (Kavita et al. 2018). The sRNA–mRNA base-pairing leads mostly to inhibition and sometimes to activation of translation of target mRNAs. In addition, Hfq interacts with several proteins including RNase E and polynucleotide phosphorylase, affecting the activities of the associated proteins. For example, the Hfq–RNase E interaction causes RNase E-dependent destabilization of the mRNAs/sRNA duplex (Massé et al. 2003; Morita et al. 2005). Hfq has been shown to mediate transcription antitermination at ρ-dependent terminators by interacting with ρ (Rabhi et al. 2011; Sedlyarova et al. 2016).

Hfq is a bacterial homolog of the eukaryotic Sm-like (LSm) proteins and forms a donut-shaped homo-hexamer (Schumacher et al. 2002; Sun et al. 2002). The Hfq hexamer has three RNA-binding surfaces: proximal face, distal face, and lateral face (rim), along with a flexible C-terminal tail (Updegrove et al. 2016). The proximal face binds the poly-uridine stretch at the 3′-end of the ρ-independent terminator of Hfq-dependent sRNAs (Otaka et al. 2011; Sauer and Weichenrieder 2011). The distal face preferentially binds the A-R(A/G)-N repeats found in the 5′-untranslated regions (5′-UTR) of many mRNAs and in certain sRNAs (Link et al. 2009; Robinson et al. 2014; Tree et al. 2014). The positively charged rim of the Hfq hexamer binds a uridine-rich internal sequence of some sRNAs and mRNAs, and has been shown to be involved in the duplex formation and RNA exchange (Panja et al. 2013; Schu et al. 2015). The binding of an sRNA and its cognate mRNA to Hfq accelerates the base-pairing between two RNAs by affecting multiple steps such as changing the structures of RNAs, bringing two RNAs into proximity, neutralizing the negative charge of two RNAs, and stimulating the annealing of two RNAs, although the actual molecular mechanism underlying for this event is not fully understood (Storz et al. 2004; Updegrove et al. 2016).

The fundamental role of Hfq in sRNA-mediated gene regulation has prompted many researchers to investigate the function and properties of Hfq. To fully understand the post-transcriptional network exerted by Hfq and its associated sRNAs, it is also important to know how the cellular concentration of Hfq is regulated. However, the regulation of Hfq synthesis has been less actively addressed and only several early studies focused on this issue. It is reported that there are about 10,000 Hfq hexamers in rapidly growing *E. coli* cells (Kajitani et al. 1994; Ali Azam et al. 1999). The level of Hfq was shown to increase at slow growth rates or at stationary phase (Tsui et al. 1997; Vytvytska et al. 1998), although other studies claimed that the level of Hfq decreases at stationary phase (Kajitani et al. 1994; Ali Azam et al. 1999). The synthesis of Hfq appears to be regulated at both transcriptional and post-transcriptional steps (Tsui et al. 1994, 1996, 1997). The *hfq* gene is part of a super-operon and its transcription is driven from several promoters including σ^32^ heat shock promoter (Tsui et al. 1996). Furthermore, it was suggested that Hfq modulates the rate of its own synthesis by causing a decrease in mRNA stability and/or translation (Tsui et al. 1997). In fact, a later study demonstrated that Hfq controls its own synthesis at the translational level by binding to the 5′-UTR of *hfq* mRNA (Vecerek et al. 2005). However, no further studies have been reported regarding the mechanism and biological significance of the autoregulation of *hfq* gene.

The aim of the present study is to gain further insights into the mechanism and the physiological significance of the *hfq* autoregulation, considering the recent progress regarding the functional and structural studies on Hfq. Here, we show that the cellular Hfq level is maintained within a limited range even when the *hfq* mRNA is overproduced in cells harboring a plasmid carrying the *hfq* gene. Then, we investigate the effects of mutations in the three RNA-binding sites of Hfq on the *hfq* autoregulation. The translational autorepression was alleviated significantly by a mutation in the distal face. We conclude that the *hfq* translation is tightly autoregulated by the binding of Hfq to the 5′-UTR of *hfq* mRNA primarily through the distal face of Hfq. The cell growth was markedly inhibited due to the increased level of Hfq caused by the distal face mutation or by mutation in the 5′-UTR of *hfq*. We further demonstrated that the rim mutation suppressed the growth inhibition caused by the distal face mutation. The physiological significance of the *hfq* autoregulation is to maintain the cellular Hfq levels within a limited range to avoid the toxicity caused by unnecessary excess Hfq.

## RESULTS

### Hfq synthesis is tightly autoregulated in vivo

**FIGURE 1.**
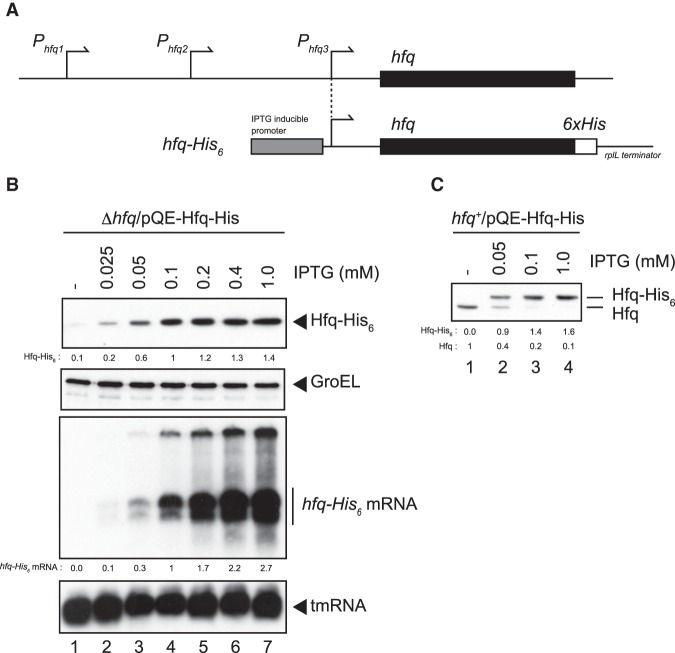
Hfq expression in cells harboring a multicopy *hfq-His*_6_ plasmid. (*A*) The *upper* diagram represents the chromosomal *hfq* region and promoters. The *hfq* gene is part of a complex operon and the transcription of *hfq* is driven from at least three promoters (Tsui et al. 1994). Two promoters (P1_*hfq*_ and P2_*hfq*_) are located in the coding region of the upstream *miaA* gene while the adjacent P3_*hfq*_ is located just upstream of the *hfq* coding region (black box). The *lower* diagram is the schematic drawing of the *hfq-His*_6_ gene on pQE-Hfq-His. The native P3_*hfq*_ is replaced with the IPTG-inducible P_*T5*_. The original 5′-UTR of *hfq* is retained in the *hfq-His*_6_. The sequence corresponding to 6xHis tag (open box) is followed by the ρ-independent terminator derived from the *rplL* (Post et al. 1979). (*B*) Expression of Hfq-His_6_ and *hfq-His*_6_ mRNA. TM589 (Δ*hfq*) cells harboring pQE-Hfq-His were grown in LB medium containing indicated concentrations of IPTG to A_600_ = ∼0.3. Total proteins and RNAs were prepared. Protein samples equivalent to 0.0125 or 0.0025 A_600_ units were subjected to western blotting using anti-His_6_ monoclonal antibody or anti-GroEL polyclonal antibodies, respectively. One μg or 0.25 μg of RNA samples was subjected to northern blotting using *hfq* or tmRNA probes, respectively. Relative Hfq-His_6_ levels and relative *hfq-His*_6_ mRNA levels are calculated, with the protein and mRNA samples at 0.1 mM IPTG set to one, respectively. (*C*) Expression of Hfq-His_6_ in wild-type cells. IT1568 cells harboring pQE-Hfq-His were grown in LB medium containing indicated concentrations of IPTG to A_600_ = ∼0.6 and total proteins were prepared. Protein samples equivalent to 0.05 A_600_ units were subjected to western blotting using anti-Hfq polyclonal antibodies. Relative Hfq-His_6_ and endogenous Hfq levels are calculated, with the endogenous Hfq level without IPTG set to one.

It was reported that the expression of Hfq is autoregulated at the translational level (Vecerek et al. 2005). However, it remains to be studied how strictly the *hfq* autoregulation operates in intact cells. We first addressed this question by using cells harboring a multicopy plasmid carrying the *hfq* gene. The plasmid pQE-Hfq-His is a derivative of pQE80L and carries the *hfq* region from the nearest promoter P3_*hfq*_ and the sequence encoding His-tag (6xHis) before the termination codon followed by a ρ-independent terminator derived from *rplL* (Post et al. 1979) at the 3′-end (Fig. 1A). We showed previously that the addition of His-tag at the C terminus of Hfq polypeptide does not affect the function of Hfq (Kawamoto et al. 2006). The *hfq-His*_6_ gene is under the control of an IPTG inducible P_*T*5_ promoter. TM589 (Δ*hfq*) cells harboring pQE-Hfq-His were grown in LB medium containing various concentrations of IPTG to mid-exponential phase. Total RNAs and proteins were prepared and analyzed by northern blotting and western blotting, respectively. The *hfq-His*_6_ mRNA and its protein product, Hfq-His_6_, were expressed in the presence of IPTG (Fig. 1B). The anti-His-tag monoclonal antibody was used to detect Hfq-His_6_ in this experiment. As expected, the *hfq-His*_6_ mRNA levels were increased with increasing concentration of IPTG up to 1 mM IPTG (Fig. 1B). Interestingly, the Hfq-His_6_ protein levels reached to a plateau at 0.1–0.2 mM IPTG. The quantitation of the bands showed that the Hfq-His_6_ level was increased only moderately (1.4-fold) from 0.1 to 1 mM IPTG, while the *hfq-His*_6_ mRNA was increased by near threefold under the same conditions (Fig. 1B, lanes 4–7). In other words, the accumulation of Hfq-His_6_ is rather moderate even when the *hfq-His*_6_ mRNA is overexpressed. Thus, the *hfq* autoregulation is quite strict to prevent overexpression of the Hfq protein.

We further investigated the *hfq* autoregulation by using IT1568 (*hfq*^+^) cells harboring pQE-Hfq-His. The anti-Hfq polyclonal antibodies were used to detect simultaneously the endogenous Hfq and Hfq-His_6_ in this experiment (Fig. 1C). When the cells were grown in the absence of IPTG, only endogenous Hfq was detected (Fig. 1C, lane 1) while Hfq-His_6_ was induced with increasing concentrations of IPTG showing again a plateau at 0.1 mM IPTG (Fig. 1C, lanes 2–4). Thus, the maximum Hfq-His_6_ level observed at 1 mM IPTG was only 1.6-fold higher than the endogenous Hfq level in the absence of IPTG. Strikingly, the level of endogenous Hfq was strongly reduced with the induction of Hfq-His_6_ (Fig. 1C, lanes 2–4). Taken together, we conclude that the *hfq* is tightly autoregulated at the translational step to maintain the cellular Hfq concentration within a limited range.

### The distal face of Hfq hexamer is responsible for the *hfq* autoregulation

**FIGURE 2.**
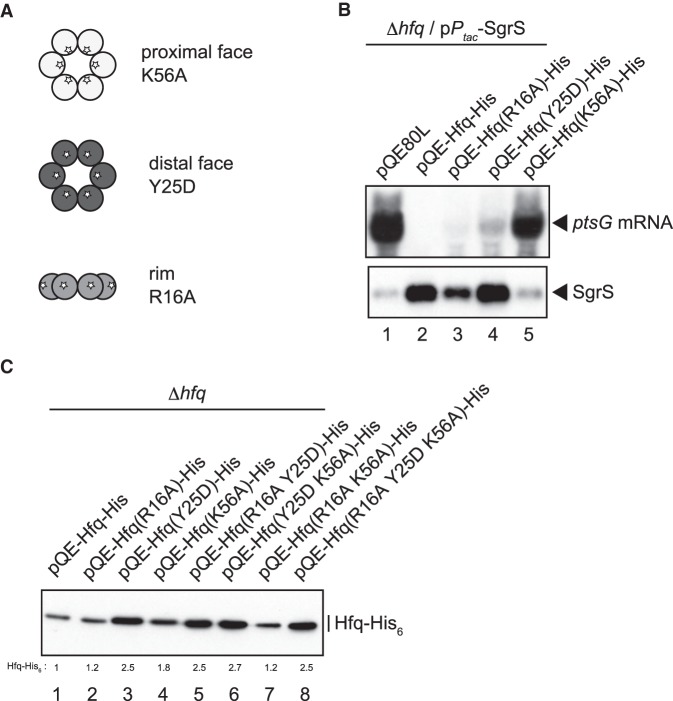
(*A*) A schematic drawing of the Hfq hexamer and location of three representative mutations on RNA-binding surfaces. (*B*) Properties of the Hfq-His_6_ derivatives carrying mutations in RNA-binding surfaces. TM589 (Δ*hfq*) cells harboring indicated plasmids were grown in LB medium containing 50 µM IPTG to A_600_ = ∼0.6 and total RNAs were prepared. Ten micrograms or 1 µg of RNA samples was subjected to northern blotting using *ptsG* probe or SgrS probe, respectively. (*C*) Effects of mutations of RNA-binding surfaces on Hfq-His_6_ expression. Overnight cultures of TM589 (Δ*hfq*) cells harboring indicated plasmids were inoculated (1/100-fold) into LB medium containing 1 mM IPTG. Incubation was continued for 90 min and then total proteins were prepared. Protein samples equivalent to 0.0125 A_600_ units were subjected to western blotting using anti-His_6_ monoclonal antibody. Relative levels of Hfq-His_6_ derivatives are calculated, with the pQE-Hfq-His sample set to one.

It was shown previously that Hfq binds to the 5′-UTR of the *hfq* mRNA to inhibit the *hfq* translation in vitro (Vecerek et al. 2005). Thus, the binding of Hfq to the *hfq* mRNA is expected to cause directly the translational inhibition of *hfq* mRNA. However, it is possible that certain sRNAs are also involved in the translational inhibition of *hfq* mRNA by Hfq. To gain further insights into the mechanism by which Hfq inhibits its own translation, we investigated the effects of mutations in the RNA-binding surfaces of Hfq on the autoregulation. The Hfq hexamer has three RNA-binding surfaces: the proximal face, distal face, and rim (Updegrove et al. 2016). To examine the roles of three RNA-binding surfaces in the *hfq* autoregulation, a series of point mutants were constructed on pQE-Hfq-His. K56A, Y25D, and R16A are representatives for Hfq variants in which the RNA-binding sites in the proximal, distal and rim faces are impaired, respectively (Zhang et al. 2013; Schu et al. 2015). The positions of these mutations on three RNA-binding surfaces of the Hfq hexamer are schematically shown in Figure 2A. The K56A variant is defective in sRNA binding, while the Y25D variant is defective in most mRNA binding (Zhang et al. 2013; Schu et al. 2015). The R16A mutation is known to modestly affect the binding of Hfq to certain sRNAs and mRNAs (Schu et al. 2015).

We first examined the effects of these mutations on the accumulation/stability of SgrS and on the ability to support the regulatory function of SgrS. Each pQE-Hfq-His variant was introduced into TM589 (Δ*hfq*) cells carrying a compatible plasmid p*P*_*tac*_-SgrS in which SgrS is constitutively expressed. Cells were grown in the presence of 0.05 mM IPTG to exponential phase and total RNAs were prepared. SgrS and *ptsG* mRNA were analyzed by northern blotting (Fig. 2B). The Hfq levels are similar to the endogenous levels in this condition. The level of SgrS was elevated in cells expressing the Hfq-His_6_, reflecting the Hfq-dependent stabilization of SgrS, resulting in the dramatic degradation of the *ptsG* mRNA (Fig. 2B, lanes 1 and 2). The results indicate that Hfq-His_6_ retains the function of wild-type Hfq. When K56A Hfq-His_6_ was expressed, SgrS did not accumulate and the rapid degradation of *ptsG* mRNA no longer occurred (Fig. 2B, lane 5), indicating that K56A Hfq-His_6_ is defective in SgrS binding; therefore it loses the ability to support the down-regulation of *ptsG* mRNA by SgrS. When Y25D Hfq-His_6_ was expressed, SgrS accumulated and the *ptsG* mRNA was partially degraded (Fig. 2B, lane 4), reflecting that the Y25D Hfq-His_6_ is defective in binding to *ptsG* mRNA but not to SgrS. The R16A mutation impacted only slightly the properties of Hfq-His_6_ (Fig. 2B, lane 3). Thus, we confirmed that the three representative Hfq-His_6_ variants possess the expected properties.

**FIGURE 3.**
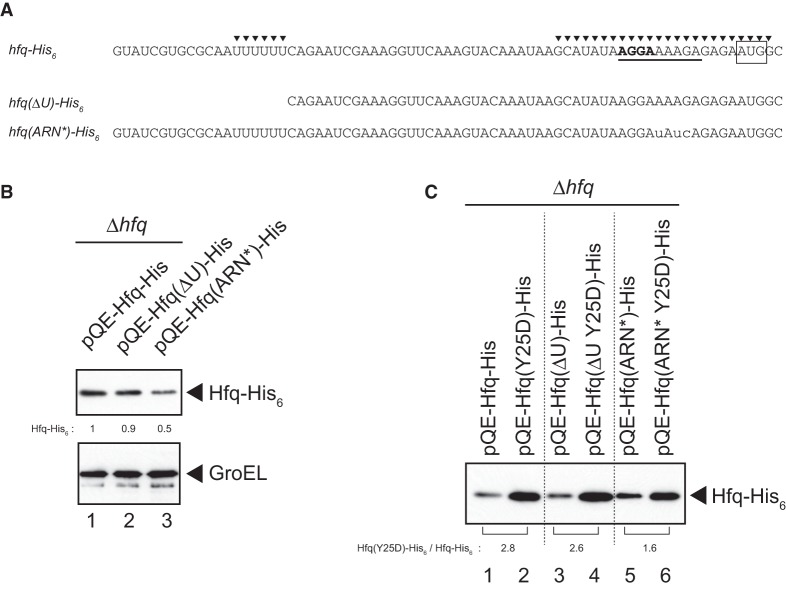
Effects of mutations in 5′-UTR on *hfq-His*_6_ expression. (*A*) RNA sequences of 5′-UTR of the wild-type and mutated *hfq* genes. The SD sequence is represented by bold letters while the initiation codon is boxed. The two Hfq-binding sites identified previously (Vecerek et al. 2005) are indicated by closed triangles. The sequence corresponding to the (ARN)_3_ repeat is underlined. The internal U-stretch along with the upstream sequence is deleted in the ΔU mutant. Three nucleotides corresponding to the (ARN)_3_ are mutated in the ARN* mutant as shown by small letters. (*B*) Overnight cultures of TM589 (Δ*hfq*) cells harboring indicated plasmids were inoculated (1/100-fold) into LB medium containing 1 mM IPTG. Incubation was continued for 90 min and then total proteins were prepared. Protein samples equivalent to 0.0125 or 0.0025 A_600_ units were subjected to western blotting using anti-His_6_ monoclonal antibody or anti-GroEL polyclonal antibodies, respectively. Relative levels of Hfq-His_6_ derivatives are calculated, with the pQE-Hfq-His sample set to one. (*C*) Protein samples mentioned above (Fig. 3B) equivalent to 0.0125 A_600_ units (lanes 1 and 2), 0.0188 A_600_ units (lanes 3 and 4), and 0.0313 A_600_ units (lanes 5 and 6) were subjected to western blotting using anti-His_6_ monoclonal antibody. The relative expression level of Hfq(Y25D)-His_6_ to Hfq-His_6_ in each 5′-UTR variant is shown *below* the gel.

Then, we investigated the effects of these mutations on the expression of Hfq-His_6_. Total proteins were prepared from cells harboring each pQE-Hfq-His variant grown in the presence of 1 mM IPTG and analyzed by western blotting (Fig. 2C). Interestingly, the Hfq-His_6_ level was significantly increased (2.5-fold) by the distal mutation Y25D (Fig. 2C, lane 3), while the protein level was moderately increased (1.8-fold) by the proximal mutation K56A (Fig. 2C, lane 4). The rim mutation R16A little affected the protein level (Fig. 2C, lane 2). We also examined the effects of the double mutations, R16A Y25D, Y25D K56A, and R16A K56A, and the triple mutation, R16A Y25D K56A, on the Hfq-His_6_ level. The Hfq-His_6_ level was increased significantly when the variants contain the Y25D substitution (Fig. 2C, lanes 5–8). Collectively, these results indicate that the distal face of Hfq plays a key role in the translational inhibition of *hfq* mRNA by Hfq. The previous work demonstrated that Hfq specifically binds two sites in the 5′-UTR of *hfq* mRNA in vitro (Vecerek et al. 2005). The downstream Hfq-binding site contains a sequence corresponding to an (ARN)_3_ repeat (Fig. 3A) that are expected to be recognized by the distal face of the Hfq hexamer. Thus, it is likely that the interaction of the Hfq hexamer with the *hfq* mRNA through the distal face is primarily responsible for the translational inhibition of *hfq* mRNA by Hfq. In addition, the K56A mutation in the proximal face also has a moderate effect on the Hfq-His_6_ level. This mutation is known to eliminate the binding of Hfq to sRNAs through the proximal face that is critical for sRNA action (Zhang et al. 2013), suggesting that undefined Hfq-dependent sRNAs are also involved, to some extent, in the translational inhibition of *hfq* mRNA by Hfq.

### The 5′-UTR of hfq is involved in the hfq autoregulation

It is known that Hfq binds two sites, the upstream site A and the downstream site B, in the 5′-UTR of *hfq* mRNA in vitro and Hfq binding to both sites is required for efficient translational repression by Hfq (Vecerek et al. 2005). Site A corresponds to the U stretch at position −55 to −50 relative to the initiation AUG codon while site B, located at position −20 to +4 encompassing the ribosome binding site, contains an (ARN)_3_ repeat (Fig. 3A). To examine the roles of these sites in the *hfq* autoregulation in vivo, we constructed two variants, pQE-Hfq(ΔU)-His and pQE-Hfq(ARN*)-His. Site A was deleted in pQE-Hfq(ΔU)-His, while the (ARN)_3_ repeat in site B was mutated in pQE-Hfq(ARN*)-His (Fig. 3A). Then, the ΔU or ARN* mutation was combined with the Y25D mutation to construct pQE-Hfq(ΔU Y25D)-His and pQE-Hfq(ARN* Y25D)-His. First, we analyzed the effect of the ΔU or ARN* mutation on the expression of Hfq-His_6_. The ARN* mutation markedly decreased the Hfq-His_6_ level while the effect of the ΔU mutation on Hfq-His_6_ expression was marginal (Fig. 3B). It is likely that the ARN* mutation reduces the efficiency of translation because the sequence around the ribosome-binding site is changed in this mutant. Then, we examined how the Y25D mutation affects the Hfq-His_6_ level in the ΔU or ARN* background. The Y25D mutation elevated the Hfq-His_6_ level by more than 2.5-fold in pQE-Hfq-His possessing the intact 5′-UTR. Importantly, the extent of increase in Hfq-His_6_ level by the Y25D mutation is significantly reduced (to 1.6-fold) in the ARN* background (Fig. 3C). The increase in Hfq-His_6_ level by the Y25D mutation appears to be little affected in the ΔU background. These results suggest that the downstream Hfq-binding site B containing the (ARN)_3_ repeat is primarily responsible for the interaction with the distal face of Hfq to achieve translational autoregulation.

### Overexpression of sRNAs alleviates the *hfq* autoregulation

**FIGURE 4.**
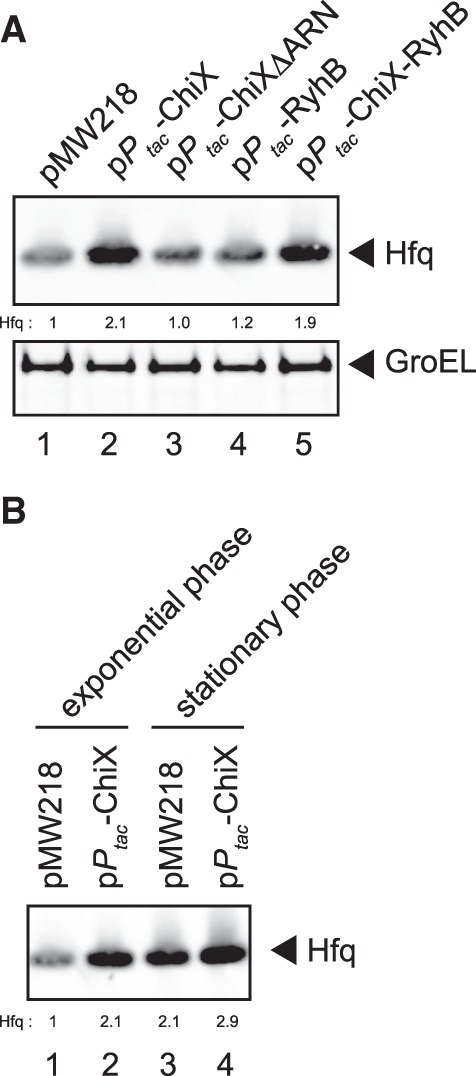
Effect of overexpression of sRNAs on Hfq expression in wild-type cells. (*A*) IT1568 cells harboring indicated plasmids were grown to A_600_ = ∼0.3. Protein samples equivalent to 0.025 or 0.0025 A_600_ units were subjected to western blotting using anti-Hfq polyclonal antibodies or anti-GroEL polyclonal antibodies, respectively. Relative Hfq levels are calculated with the pMW218 sample set to one. (*B*) IT1568 cells harboring indicated plasmids were grown to A_600_ = ∼0.3, and to A_600_ = ∼1.9. Protein samples equivalent to 0.025 A_600_ units were subjected to western blotting using anti-Hfq polyclonal antibodies. Relative Hfq levels are calculated, with the pMW218 sample at exponential phase set to one.

We expect that RNAs possessing a high affinity to Hfq compete with the *hfq* mRNA regarding Hfq binding resulting in alleviation of the autoregulation. If this is the case, the Hfq level derived from the endogenous *hfq* gene may increase when these RNAs are overexpressed. To test this, we examined the effect of overproduction of RyhB or ChiX on the endogenous Hfq protein level. RyhB is a representative of class I sRNAs that bind to the proximal and rim surfaces of the Hfq hexamer through the polyU tail and internal U-rich sequence, respectively. ChiX, an efficient Hfq titrator (Mandin and Gottesman 2010; Ellis et al. 2015), belongs to class II sRNAs that bind to the proximal and distal faces of the Hfq hexamer through the polyU tail and internal ARN repeats, respectively (Schu et al. 2015). These sRNA genes were placed under the *P*_*tac*_ constitutive promoter on a plasmid. Each plasmid was introduced into IT1568 (*hfq*^+^) and cells were grown to exponential phase. Total proteins were subjected to western blotting using anti-Hfq antibodies. As shown in Figure 4A, the Hfq protein level was significantly (2.1-fold) increased when ChiX was overexpressed (Fig. 4A, lane 2). The effect of RyhB overproduction was less significant (Fig. 4A, lane 4). The results suggest that ChiX effectively competes with the *hfq* mRNA resulting in alleviation of the *hfq* autoregulation primarily through the ARN repeat. If so, the competition by ChiX would be impaired when its ARN repeat is removed. In fact, we found that overexpression of the ChiX derivative lacking the ARN repeat, ChiXΔARN (Schu et al. 2015), did not affect the level of endogenous Hfq protein (Fig. 4A, lane 3). We also found that the Hfq protein level was significantly increased when the RyhB derivative containing the ARN repeat derived from ChiX, ChiX-RyhB (Schu et al. 2015), was overexpressed (Fig. 4A, lane 5). Taken together, we conclude that class II sRNAs efficiently compete with the *hfq* mRNA through the interaction of their internal ARN repeats with the distal face of Hfq to alleviate the *hfq* autoregulation. In this regard, it should be noted that the increase of Hfq level, though derived from the plasmid-borne *hfq* gene, by overproduction of OxyS was previously observed (Moon and Gottesman 2011). It is known that the level of endogenous Hfq increases when cells enter into stationary phase (Tsui et al. 1997; Vytvytska et al. 1998). We confirmed this by showing that the Hfq level increases by about twofold in stationary phase (Fig. 4B, lanes 1 and 3). We also found that ChiX overproduction still affects the Hfq level though less significantly at stationary phase (Fig. 4B, lanes 3 and 4). The results suggest that the increase of Hfq at stationary phase would be achieved at least in part through the alleviation of the *hfq* autoregulation by RNAs containing ARN repeats including sRNAs generated during stationary phase.

### Effects of Hfq levels and Hfq mutations on cell growth

**FIGURE 5.**
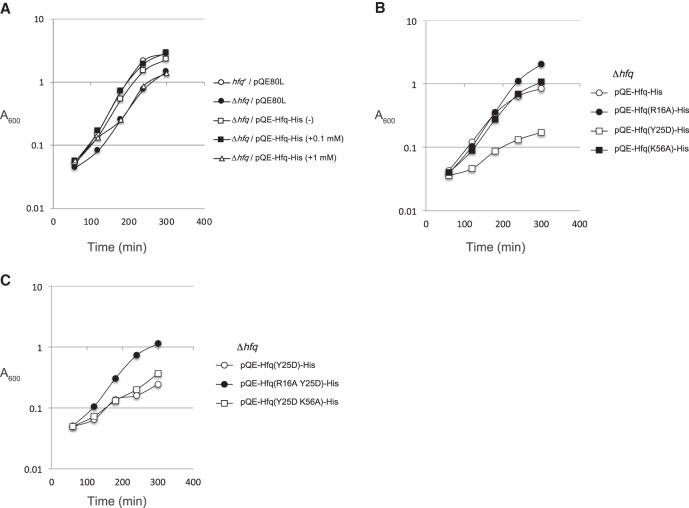
(*A*) Effect of expression of the plasmid borne *hfq-His*_6_ gene on cell growth. Overnight cultures of TM589 (Δ*hfq*) cells harboring indicated plasmids were inoculated (1/1000-fold) into LB medium supplemented with indicated concentrations of IPTG. Incubation was continued for 300 min by measuring A_600_. (*B*) Effect of expression of the plasmid borne mutated *hfq-His*_6_ gene on cell growth. Overnight cultures of TM589 (Δ*hfq*) cells harboring indicated plasmids were inoculated (1/1000-fold) into LB medium supplemented with 1 mM IPTG. Incubation was continued for 300 min by measuring A_600_. (*C*) The rim mutation suppresses growth inhibition caused by the distal mutation. Overnight cultures of TM589 (Δ*hfq*) cells harboring indicated plasmids were inoculated (1/1000-fold) into LB medium supplemented with 1 mM IPTG. Incubation was continued for 300 min by measuring A_600_.

To explore the physiological significance of the *hfq* autoregulation, we analyzed the effect of expression of the plasmid borne *hfq* on cell growth. IT1568 (*hfq*^+^) and TM589 (Δ*hfq*) cells harboring pQE-Hfq-His or the vector pQE80L were grown in LB medium supplemented with different concentrations of IPTG. It is known that an *hfq* insertion mutant displays a decreased growth rate compared with the *hfq*^+^ strain (Tsui et al. 1994). We confirmed this early observation by showing that the Δ*hfq* cells harboring pQE80L grow slowly compared with the *hfq*^+^ cells harboring pQE80L (Fig. 5A, open and closed circles). The growth of Δ*hfq* cells harboring pQE-Hfq-His is better than that of Δ*hfq* cells harboring pQE80L (Fig. 5A, open square). This is consistent with the observation that Hfq-His is expressed, though at a lower level, even without IPTG in cells harboring pQE-Hfq-His (Fig. 1B). The addition of 0.1 mM IPTG to Δ*hfq* cells harboring pQE-Hfq-His restored almost completely the growth (Fig. 5A, closed square). The level of Hfq-His_6_ in Δ*hfq* cells in this condition is slightly higher than the endogenous Hfq level in the *hfq*^+^ cells (Fig. 1B,C). Interestingly, the growth of Δ*hfq* cells harboring pQE-Hfq-His was slightly inhibited when 1 mM IPTG was added (Fig. 5A, open triangle). In this condition, the Hfq-His_6_ level is higher by only 1.6-fold compared with the endogenous Hfq level in the *hfq*^+^ cells, while the *hfq* mRNA is highly overexpressed (Fig. 1B). This implies that Hfq-His_6_ accumulates beyond the wild-type Hfq level by partially escaping the autoregulation when the *hfq-His*_6_ mRNA is overexpressed. This moderate increase of Hfq-His_6_ level may lead to the moderate growth inhibition of Δ*hfq* cells harboring pQE-Hfq-His. We expected that the distal face mutation Y25D would lead to a more severe growth inhibition because the Hfq-His_6_ level is further increased in cells harboring pQE-Hfq(Y25D)-His under IPTG induction (Fig. 2B). Indeed, the cell growth was markedly inhibited when the *hfq-His*_6_ gene containing the Y25D variant was expressed by the addition of 1 mM IPTG (Fig. 5B, open square). On the other hand, the introduction of proximal mutation K56A into the *hfq-His*_6_ gene increased Hfq-His level only moderately and little affected the cell growth (Figs. 2B, 5B, closed square). Interestingly, the growth became apparently better when the R16A rim mutation was introduced (Fig. 5B, closed circle). This suggests that the rim is somehow involved in the growth inhibition by increased Hfq levels.

### Increased Hfq level leads to inhibition of cell growth

**FIGURE 6.**
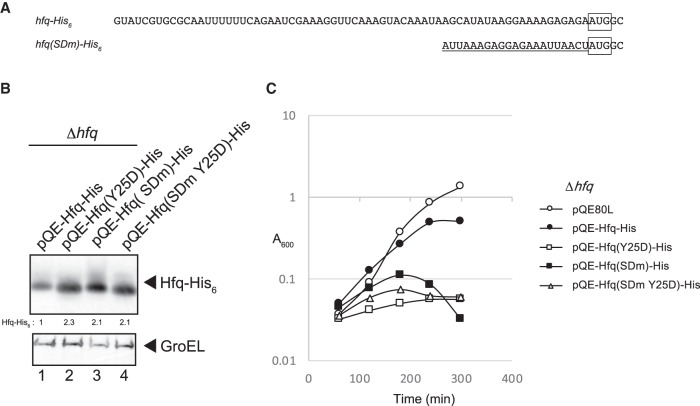
Effects of replacement of the 5′-UTR of *hfq* mRNA with an unrelated 5′-UTR. (*A*) RNA sequences of 5′-UTR of the wild-type and the mutated *hfq* gene. The initiation codon is boxed and the sequence corresponding to the SD sequence derived from the plasmid pQE80L is underlined. (*B*) Overnight cultures of TM589 (Δ*hfq*) cells harboring indicated plasmids were inoculated (1/100-fold) into LB medium containing 1 mM IPTG. Incubation was continued for 60 min and then total proteins were prepared. Protein samples equivalent to 0.0063 or 0.0025 A_600_ units were subjected to western blotting using anti-His_6_ monoclonal antibody or anti-GroEL polyclonal antibodies, respectively. Relative levels of Hfq-His_6_ derivatives are calculated, with the pQE-Hfq-His sample set to one. (*C*) Effect of expression of the wild-type and mutated *hfq-His*_6_ genes on cell growth. Overnight cultures of TM589 (Δ*hfq*) cells harboring indicated plasmids were inoculated (1/1000-fold) into LB medium supplemented with 1 mM IPTG. Incubation was continued for 300 min by measuring A_600_.

The results mentioned above suggest that the distal face mutation Y25D leads to the severe growth inhibition presumably by increasing the Hfq level. To examine this, we constructed plasmids pQE-Hfq(SDm)-His and pQE-Hfq(SDm Y25D)-His in which the *hfq* 5′-UTR was replaced with the 5′-UTR containing SD sequence derived from the plasmid pQE (Fig. 6A). These plasmids along with pQE-Hfq-His and pQE-Hfq(Y25D)-His were individually introduced into TM589 (Δ*hfq*) cells. Cells were grown under IPTG induction and total proteins were analyzed by western blotting. The Hfq-His protein level was increased about by 2.1-fold of the SDm mutation (Fig. 6B, lane 3). This increase of Hfq level is comparable to that by the Y25D mutation (Fig. 6B, lanes 2 and 4). Then, we examined the effect of expression of Hfq-His and Hfq(Y25D)-His on cell growth. The growth of cells harboring pQE-Hfq(SDm)-His was significantly inhibited in the presence of IPTG (Fig. 6C). This is consistent with the view that a significant increase in the Hfq protein level beyond the wild-type level is harmful to cell growth. It should be noted, however, that the patterns of growth inhibition by pQE-Hfq(SDm)-His and pQE-Hfq(Y25D)-His are clearly different each other. The pQE-Hfq(Y25D)-His inhibits the cell growth more strongly at the initial stage of growth, suggesting that the unique nature of HfqY25D protein itself may contribute to the toxicity by pQE-Hfq(Y25D)-His (Fig. 6C). In addition, pQE-Hfq(SDm)-His but not pQE-Hfq(Y25D)-His or pQE-Hfq(SDm Y25D)-His leads to cell lysis finally (Fig. 6C). This suggests that the distal face of wild-type Hfq is somehow involved in cells lysis.

### A mutation in the rim suppresses the growth inhibition caused by the distal mutation

Because the rim mutation improves the growth inhibition caused by the modest increase of the wild-type Hfq-His, it is interesting to examine the effect of the rim mutation on the severe growth inhibition caused by the Y25D distal mutation. Therefore, we examined the cell growth of R16A Y25D and Y25D K56A double mutants when the *hfq-His*_6_ gene harboring each double mutation was expressed in the presence of 1 mM IPTG. Interestingly, the R16A rim mutation greatly suppressed the growth inhibition caused by the Y25D distal mutation (Fig. 5C, closed circle). On the other hand, the growth inhibition caused by the Y25D mutation was not affected by introducing the K56A proximal mutation (Fig. 5C, open square). It should be noted that the increased Hfq-His_6_ level due to Y25D mutation is not affected by the second R16A or K56A mutation (Fig. 2B). Thus, the suppression of growth inhibition by R16A is not due to the reduction of Hfq-His_6_ level. These results suggest that the interaction of excess Hfq with undefined RNAs through the rim is responsible for the growth inhibition by increased Hfq levels.

## DISCUSSION

It was shown previously that the *hfq* translation is repressed by Hfq (Vecerek et al. 2005). In the present study, we investigated the detailed mechanism and physiological significance of the *hfq* autoregulation. We first addressed a question of how strictly the *hfq* autoregulation operates in cells. We showed that the concentration of Hfq-His_6_ is increased only modestly even when the *hfq-His*_6_ mRNA is overproduced in Δ*hfq* cells harboring the *hfq-His*_6_ gene on a multicopy plasmid (Fig. 1A,B). The maximum level of Hfq-His_6_ is less than twofold compared with the endogenous Hfq level in *hfq*^+^ cells (Fig. 1C). Hfq-His_6_ expressed from the plasmid strongly inhibits the Hfq synthesis from the chromosomal *hfq* gene (Fig. 1C). We conclude that the translational repression by Hfq is operating quite strictly in vivo to maintain the cellular Hfq concentration in a limited range.

The inhibition of *hfq* translation by Hfq can be achieved by two different mechanisms. Firstly, Hfq could inhibit the *hfq* translation by binding to the 5′-UTR of *hfq* mRNA in the absence of any sRNA. In this case, Hfq acts directly as a translational repressor for the *hfq* gene. Secondly, Hfq could inhibit the *hfq* translation by supporting the action of Hfq-dependent base-pairing sRNAs. In this case, the base-pairing itself rather than Hfq is responsible for the translational repression (Maki et al. 2008). The major conclusion in the present study is that the interaction of the Hfq hexamer with the 5′-UTR of *hfq* mRNA through the distal face is primarily responsible for the translational inhibition of *hfq* mRNA by Hfq. We demonstrated that the distal face Y25D mutation of the Hfq hexamer markedly alleviates the tight repression of Hfq synthesis by Hfq, while the proximal face K56A mutation does not have a strong effect on the autoregulation (Fig. 2B). Interestingly, it is observed that the cellular Hfq level is significantly increased by the Y25D mutation in the chromosomal *hfq* gene (Chen and Gottesman 2017).

The *hfq* autoregulation was the first example for translational repression by direct binding of Hfq to the 5′-UTR of the target mRNA (Vecerek et al. 2005). Recent studies have demonstrated that there are several cases in which Hfq acts on mRNAs to inhibit translation in an sRNA-independent manner. For example, the translation of Tn10 transposase mRNA is repressed by Hfq binding at the ribosome binding site that directly blocks ribosome entry (Ellis et al. 2015). Similarly, Hfq was shown to bind near ribosome binding sites to inhibit the translation of *cirA* mRNA (Salvail et al. 2013) and *amiE* mRNA in *Pseudomonas aeruginosa* (Sonnleitner and Bläsi 2014). More recently, it was shown that Hfq inhibits the translation by binding to the 5′-UTR of *mutS* mRNA (Chen and Gottesman 2017). In this case, Hfq binds a specific site containing ARN repeats located upstream of the ribosome-binding site presumably by altering the RNA structure resulting in translational repression. In addition, Hfq-dependent sRNAs are also involved in the translational inhibition of *mutS* by Hfq (Chen and Gottesman 2017).

Our mutational study supports the previous view (Vecerek et al. 2005) that the 5′-UTR of the *hfq* is required for the autoregulation (Figs. 2B, 3C). The binding of Hfq to the downstream site B through the distal face of the Hfq hexamer is expected to directly block ribosome access. The role of Hfq binding at the upstream site A is not clear at this moment though it could contribute to the translational inhibition by changing the RNA structure as in the case of *mutS* (Chen and Gottesman 2017). It remains to be studied which RNA-binding surface is involved in Hfq binding to the upstream U-rich stretch and how the interaction between Hfq and the U-rich sequence contributes to the translational repression of the *hfq* by Hfq. In any case, it is apparent that Hfq represses its own translation by binding the 5′-region of the *hfq* mRNA primarily through the distal face. This conclusion is supported by the finding that overexpression of an sRNA possessing ARN repeats significantly increases the endogenous Hfq levels (Fig. 4A). We also showed that the proximal face K56A mutation moderately alleviates the repression of Hfq synthesis by Hfq (Fig. 2B). This suggests that undefined sRNAs are also involved in the *hfq* autoregulation in part as observed in the translational repression of *mutS* by Hfq (Chen and Gottesman 2017) because the action of Hfq-dependent sRNAs requires the interaction between the proximal face of Hfq and the polyU tail of sRNAs (Otaka et al. 2011). In fact, IntaRNA (Freiburg RNA tools) analysis of the RIL-seq data (Melamed et al. 2016) suggest that several sRNAs including 3′-UTR of *ibpB* are partially complementary to the translation initiation region of the *hfq* mRNA. It is certainly interesting to examine experimentally whether these sRNAs are involved in the *hfq* autoregulation.

We focused here on the translational inhibition of *hfq* mRNA by Hfq as the major mechanism of the *hfq* autoregulation. However, we do not exclude the possibility that destabilization of *hfq* mRNA by Hfq also contributes to the *hfq* autoregulation as suggested in an early study (Tsui et al. 1997). In fact, we demonstrated previously that Hfq interacts with a specific site within the C-terminal region of RNase E resulting in RNase E-dependent rapid degradation of target mRNAs of Hfq-binding sRNAs (Morita et al. 2005; Ikeda et al. 2011). It is an interesting possibility that Hfq destabilizes the *hfq* mRNA through the direct Hfq binding to *hfq* mRNA. Studies on how Hfq affects the stability of its own mRNA are certainly important to fully understand the molecular mechanism of the *hfq* autoregulation.

It is known that the Hfq level increases at slow growth rates or at stationary phase (Tsui et al. 1997; Vytvytska et al. 1998). However, the mechanism by which the Hfq level varies depending on the growth phase is totally unknown. The present study has shed a light on this question. We demonstrated that overexpression of ChiX containing ARN repeats significantly increases the Hfq level in wild-type cells at exponential phase (Fig. 4B). An intriguing idea is that the increase of Hfq at stationary phase would be achieved at least in part by the alleviation of the *hfq* autoregulation by RNAs containing ARN repeats including sRNAs generated during stationary phase. In this regard, it is interesting to note that some sRNAs including ChiX (SroB) seem to be more expressed at stationary phase (Vogel et al. 2003; Zhang et al. 2003). We believe that the translational repression of *hfq* mRNA by Hfq operates not only in cells harboring a multicopy *hfq* gene but also in the wild-type cells. Further studies are necessary to fully elucidate the mechanism by which Hfq level varies depending on the physiological status.

Cells lacking the *hfq* gene are known to grow slowly compared to *hfq*^+^ cells (Tsui et al. 1994). We showed here that the moderate elevation of Hfq level by overexpression of *hfq* mRNA also negatively affects cell growth (Fig. 5A). The cell growth is markedly inhibited when the Hfq level is further increased by the distal mutation (Fig. 5B) or by replacing the *hfq* 5′-UTR with an unrelated SD sequence (Fig. 6). Thus, it is apparent that excess cellular Hfq is toxic to cells. However, the inhibitory effect of excess HfqY25D protein is stronger compared to excess wild-type Hfq at the initial stage of growth, suggesting that the unique nature of HfqY25D protein also may contribute to the toxicity (Fig. 6). Another difference between two proteins is that wild-type Hfq but not HfqY25D leads to cell lysis finally. The distal face of Hfq is apparently responsible for cell lysis under excess expression of wild-type Hfq. The physiological significance of the *hfq* autoregulation would be to prevent unnecessary increase of Hfq resulting in an optimum Hfq level in a given physiological condition. An additional interesting finding is that the growth inhibition caused by the Y25D mutation is suppressed by a rim mutation (Fig. 5C). This suggests that the interaction of excess Hfq with certain cellular RNAs through the rim are harmful to cells. The identification of such RNAs would be certainly interesting and should contribute to understanding why the excess Hfq is toxic to cells.

## MATERIALS AND METHODS

### Bacterial strains and plasmids

The *E. coli* K12 strains and plasmids used in this study are listed in Table 1. The DNA primers used for the plasmid construction are listed in Table 2. To construct pQE-Hfq-His, the *hfq-His*_6_ sequence was amplified with primers 1450 and 1451 using genomic DNA derived from W3110*mlc* as a DNA template. The amplified DNA fragment was digested with *Eco*RI and *Hin*dIII and cloned into pQE80L. A series of derivatives of pQE-Hfq-His in which one or more of the three RNA-binding surfaces of Hfq are mutated were constructed as follows. DNA fragment 1 containing the *hfq(R16A)-His*_6_ sequence was amplified with primers 1450 and 1457 using pQE-Hfq-His as a DNA template. DNA fragment 2 containing the *hfq(R16A)-His*_6_ sequence was similarly amplified with primers 1451 and 1456. Then, the DNA fragments 1 and 2 were used to amplify the entire *hfq(R16A)-His*_6_ region with primers 1450 and 1451. The amplified DNA fragment was digested with *Eco*RI and *Hin*dIII and cloned into pQE80L to construct pQE-Hfq(R16A)-His. Similarly, pQE-Hfq(Y25D)-His and pQE-Hfq(K56A)-His were constructed by using the corresponding DNA primers. The pQE-Hfq-His derivatives carrying the double and triple mutations were constructed by using the corresponding DNA primers and pQE-Hfq-His derivatives as DNA templates. Plasmids pQE-Hfq(ΔU)-His and pQE-Hfq(Y25D ΔU)-His were constructed as follows: pQE-Hfq-His and pQE-Hfq (Y25D)-His were used to amplify the *hfq(*Δ*U)-His*_6_ and *hfq (Y25D*Δ*U)-His*_6_ sequences with primers 2020 and 1451, respectively. The amplified DNA fragments were digested with *Eco*RI and *Hin*dIII and cloned into pQE80L. Similarly, pQE-Hfq(ARN*)-His and pQE-Hfq(Y25D ARN*)-His were constructed by using primers 2019 and 1451. Plasmids p*P*_*tac*_-RyhB, p*P*_*tac*_-SgrS, and p*P*_*tac*_-ChiX were constructed as follows: pRyhB, pSgrS (Otaka et al. 2011), and chromosomal DNA of W3110*mlc* were used to amplify the DNA fragment containing *ryhB*, *sgrS*, and *chiX* sequence with primers 2011/1145, 2009/1839, and 1978/1979, respectively. The amplified DNA fragments were digested with *Eco*RI and *Hin*dIII and cloned into pMW218. Plasmids p*P*_*tac*_-ChiXΔARN, p*P*_*tac*_-ChiX-RyhB, pQE-Hfq(SDm)-His, and pQE-Hfq(SDm Y25D)-His were constructed by in vitro recombination using the In-Fusion HD Cloning kit (Takara Bio USA). p*P*_*tac*_-ChiX and p*P*_*tac*_-RyhB were used to amplify the DNA fragment containing *chiX*Δ*ARN* and *chiX-ryhB* sequence with primers 2039/2040 and 2041/2042, respectively. pQE-Hfq-His and pQE-Hfq(Y25D)-His were used to amplify the DNA fragment containing *hfq(SDm)-His* and *hfq(SDm Y25D)-His* sequence with primers 2043/2044, respectively.

**TABLE 1.**
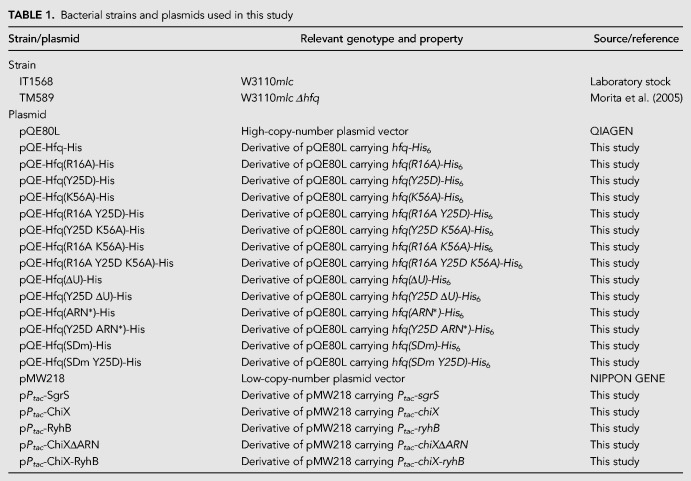
Bacterial strains and plasmids used in this study

**TABLE 2.**
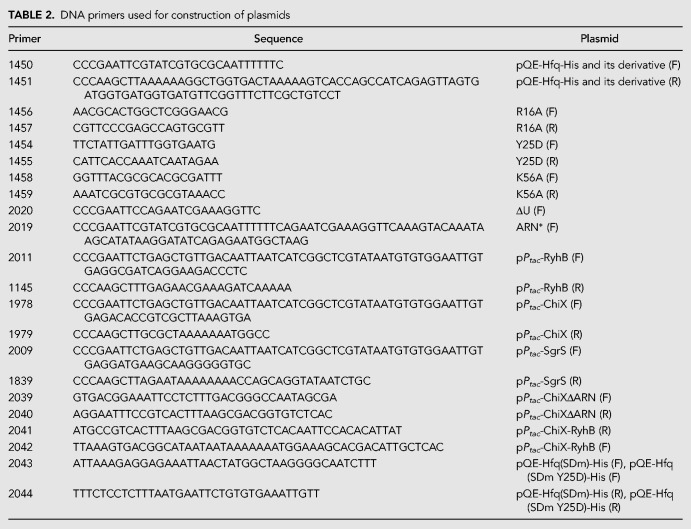
DNA primers used for construction of plasmids

### Media and growth condition

Cells carrying indicated plasmids were grown at 37°C in LB medium supplemented with ampicillin (50 µg/mL) and/or kanamycin (15 µg/mL) when necessary. Overnight cultures were diluted 1000-fold (for growth experiments) or 100-fold (for protein or RNA analyses) into the same fresh medium supplemented with indicated concentrations of IPTG. Cell growth was monitored by determining the absorbance at 600 nm.

### Western blotting

The cultures (500 µL) were centrifuged and the cell pellets were suspended in SDS-PAGE loading buffer (6.25 mM Tris-HCl at pH 6.8, 2% SDS, 10% glycerol, 5% β-mercaptoethanol, 0.1% bromophenol blue). The sample was heated for 5 min at 100°C and subjected to a 15% (for Hfq and Hfq-His_6_) or 10% (for GroEL) polyacrylamide-0.1% SDS gel electrophoresis and transferred to an Immobilon membrane (Millipore). The membranes were treated either with anti-Hfq polyclonal antibodies (Morita et al. 2005), an anti-His_6_ monoclonal antibody (Wako), and anti-GroEL polyclonal antibodies (MERCK). Signals were visualized by the Lumi-Light Western Blotting Substrate (Merck). Multi Gauge ver. 3.1 software (Fujifilm) was used to quantify protein bands on the films. In terms of Figures 4 and 6B, the culture (1 mL) was centrifuged and pellets were suspended in LDS loading buffer (Invitrogen). The sample was heated for 10 min at 95°C, subjected to a 12% Bis-Tris gel electrophoresis (Invitrogen), and transferred to a nitrocellulose membrane (Invitrogen). The membranes were treated either with anti-Hfq polyclonal antibodies (Moon and Gottesman 2011), an anti-His_6_ monoclonal antibody (Wako), and anti-GroEL polyclonal antibodies (MERCK). StarBright Blue 700 Goat Anti-Rabbit IgG (BioRad) and DyLight800 Goat Anti-Mouse IgG (BioRad) were used as secondary antibodies for detection in fluorescence western blotting. Images were obtained with a ChemiDoc MP imaging system (BioRad). ImageQuant TL software (GE Healthcare) was used to quantify protein bands.

### Northern blotting

Total RNAs were isolated as described by Aiba et al. (1981). RNA samples were resolved by 1.5% (for *hfq-His*_6_ mRNA, tmRNA, and SgrS) or 1.2% (for *ptsG* mRNA) agarose gel electrophoresis in the presence of formaldehyde and blotted onto Hybond-N^+^ membrane (GE healthcare). The RNAs were visualized using digoxigenin (DIG) reagents and kits for nonradioactive nucleic acid labeling and a detection system (MERCK) according to the procedure specified by the manufacturer. The following DIG-labeled DNA probes were prepared by PCR using DIG-dUTP: a 309-bp fragment corresponding to the coding region of *hfq* (*hfq* probe); 363-bp fragment corresponding to the tmRNA (tmRNA probe); 305-bp fragment corresponding to the 5′ region of *ptsG* (*ptsG* probe); 227-bp fragment corresponding to the *sgrS* (SgrS probe). Multi Gauge Ver. 3.1 software (Fujifilm) was used to quantify RNA bands on the films.

## References

[RNA068106MORC1] Aiba H, Adhya S, de Crombrugghe B. 1981 Evidence for two functional *gal* promoters in intact *Escherichia coli* cells. J Biol Chem 256: 11905–11910. 6271763

[RNA068106MORC2] Ali Azam T, Iwata A, Nishimura A, Ueda S, Ishihama A. 1999 Growth phase-dependent variation in protein composition of the *Escherichia coli* nucleoid. J Bacteriol 181: 6361–6370. 10515926 10.1128/jb.181.20.6361-6370.1999 PMC103771

[RNA068106MORC3] Chen J, Gottesman S. 2017 Hfq links translation repression to stress-induced mutagenesis in *E. coli*. Genes Dev 31: 1382–1395. 10.1101/gad.302547.117 28794186 PMC5580658

[RNA068106MORC4] Ellis MJ, Trussler RS, Haniford DB. 2015 Hfq binds directly to the ribosome-binding site of IS10 transposase mRNA to inhibit translation. Mol Microbiol 96: 633–650. 10.1111/mmi.12961 25649688 PMC5006887

[RNA068106MORC5] Franze de Fernandez MT, Eoyang L, August JT. 1968 Factor fraction required for the synthesis of bacteriophage Qβ-RNA. Nature 219: 588–590. 10.1038/219588a0 4874917

[RNA068106MORC6] Ikeda Y, Yagi M, Morita T, Aiba H. 2011 Hfq binding at RhlB-recognition region of RNase E is crucial for the rapid degradation of target mRNAs mediated by sRNAs in *Escherichia coli*. Mol Microbiol 79: 419–432. 10.1111/j.1365-2958.2010.07454.x 21219461

[RNA068106MORC7] Kajitani M, Kato A, Wada A, Inokuchi Y, Ishihama A. 1994 Regulation of the *Escherichia coli hfq* gene encoding the host factor for phage Q_β_. J Bacteriol 176: 531–534. 10.1128/jb.176.2.531-534.1994 8288550 PMC205081

[RNA068106MORC8] Kavita K, de Mets F, Gottesman S. 2018 New aspects of RNA-based regulation by Hfq and its partner sRNAs. Curr Opin Microbiol 42: 53–61. 10.1016/j.mib.2017.10.014 29125938 PMC10367044

[RNA068106MORC9] Kawamoto H, Koide Y, Morita T, Aiba H. 2006 Base-pairing requirement for RNA silencing by a bacterial small RNA and acceleration of duplex formation by Hfq. Mol Microbiol 61: 1013–1022. 10.1111/j.1365-2958.2006.05288.x 16859494

[RNA068106MORC10] Link TM, Valentin-Hansen P, Brennan RG. 2009 Structure of *Escherichia coli* Hfq bound to polyriboadenylate RNA. Proc Natl Acad Sci 106: 19292–19297. 10.1073/pnas.0908744106 19889981 PMC2773200

[RNA068106MORC11] Maki K, Uno K, Morita T, Aiba H. 2008 RNA, but not protein partners, is directly responsible for translational silencing by a bacterial Hfq-binding small RNA. Proc Natl Acad Sci 105: 10332–10337. 10.1073/pnas.0803106105 18650387 PMC2492515

[RNA068106MORC12] Mandin P, Gottesman S. 2010 Integrating anaerobic/aerobic sensing and the general stress response through the ArcZ small RNA. EMBO J 29: 3094–3107. 10.1038/emboj.2010.179 20683441 PMC2944060

[RNA068106MORC13] Massé E, Escorcia FE, Gottesman S. 2003 Coupled degradation of a small regulatory RNA and its mRNA targets in *Escherichia coli*. Genes Dev 17: 2374–2383. 10.1101/gad.1127103 12975324 PMC218075

[RNA068106MORC14] Melamed S, Peer A, Faigenbaum-Romm R, Gatt YE, Reiss N, Bar A, Altuvia Y, Argaman L, Margalit H. 2016 Global mapping of small RNA-target interactions in bacteria. Mol Cell 63: 884–897. 10.1016/j.molcel.2016.07.026 27588604 PMC5145812

[RNA068106MORC15] Moon K, Gottesman S. 2011 Competition among Hfq-binding small RNAs in *Escherichia coli*. Mol Microbiol 82: 1545–1562. 10.1111/j.1365-2958.2011.07907.x 22040174 PMC7394283

[RNA068106MORC16] Morita T, Maki K, Aiba H. 2005 RNase E-based ribonucleoprotein complexes: mechanical basis of mRNA destabilization mediated by bacterial noncoding RNAs. Genes Dev 19: 2176–2186. 10.1101/gad.1330405 16166379 PMC1221888

[RNA068106MORC17] Morita T, Ueda M, Kubo K, Aiba H. 2015 Insights into transcription termination of Hfq-binding sRNAs of *Escherichia coli* and characterization of readthrough products. RNA 21: 1490–1501. 10.1261/rna.051870.115 26106215 PMC4509938

[RNA068106MORC18] Morita T, Nishino R, Aiba H. 2017 Role of the terminator hairpin in the biogenesis of functional Hfq-binding sRNAs. RNA 23: 1419–1431. 10.1261/rna.060756.117 28606943 PMC5558911

[RNA068106MORC19] Otaka H, Ishikawa H, Morita T, Aiba H. 2011 PolyU tail of ρ-independent terminator of bacterial small RNAs is essential for Hfq action. Proc Natl Acad Sci 108: 13059–13064. 10.1073/pnas.1107050108 21788484 PMC3156202

[RNA068106MORC20] Panja S, Schu DJ, Woodson SA. 2013 Conserved arginines on the rim of Hfq catalyze base pair formation and exchange. Nucleic Acids Res 41: 7536–7546. 10.1093/nar/gkt521 23771143 PMC3753642

[RNA068106MORC21] Post LE, Strycharz GD, Nomura M, Lewis H, Dennis PP. 1979 Nucleotide sequence of the ribosomal protein gene cluster adjacent to the gene for RNA polymerase subunit β in *Escherichia coli*. Proc Natl Acad Sci 76: 1697–1701. 10.1073/pnas.76.4.1697 377281 PMC383457

[RNA068106MORC22] Rabhi M, Espéli O, Schwartz A, Cayrol B, Rahmouni AR, Arluison V, Boudvillain M. 2011 The Sm-like RNA chaperone Hfq mediates transcription antitermination at ρ-dependent terminators. EMBO J 30: 2805–2816. 10.1038/emboj.2011.192 21673658 PMC3160251

[RNA068106MORC23] Robinson KE, Orans J, Kovach AR, Link TM, Brennan RG. 2014 Mapping Hfq-RNA interaction surfaces using tryptophan fluorescence quenching. Nucleic Acids Res 42: 2736–2749. 10.1093/nar/gkt1171 24288369 PMC3936774

[RNA068106MORC24] Salvail H, Caron MP, Bélanger J, Massé E. 2013 Antagonistic functions between the RNA chaperone Hfq and an sRNA regulate sensitivity to the antibiotic colicin. EMBO J 32: 2764–2778. 10.1038/emboj.2013.205 24065131 PMC3801439

[RNA068106MORC25] Sauer E, Weichenrieder O. 2011 Structural basis for RNA 3′-end recognition by Hfq. Proc Natl Acad Sci 108: 13065–13070. 10.1073/pnas.1103420108 21737752 PMC3156190

[RNA068106MORC26] Schu DJ, Zhang A, Gottesman S, Storz G. 2015 Alternative Hfq-sRNA interaction modes dictate alternative mRNA recognition. EMBO J 34: 2557–2573. 10.15252/embj.201591569 26373314 PMC4609186

[RNA068106MORC27] Schumacher MA, Pearson RF, Møller T, Valentin-Hansen P, Brennan RG. 2002 Structures of the pleiotropic translational regulator Hfq and an Hfq-RNA complex: a bacterial Sm-like protein. EMBO J 21: 3546–3556. 10.1093/emboj/cdf322 12093755 PMC126077

[RNA068106MORC28] Sedlyarova N, Shamovsky I, Bharati BK, Epshtein V, Chen J, Gottesman S, Schroeder R, Nudler E. 2016 sRNA-mediated control of transcription termination in *E. coli*. Cell 167: 111–121.e113. 10.1016/j.cell.2016.09.004 27662085 PMC5040353

[RNA068106MORC29] Sobrero P, Valverde C. 2012 The bacterial protein Hfq: much more than a mere RNA-binding factor. Crit Rev Microbiol 38: 276–299. 10.3109/1040841X.2012.664540 22435753

[RNA068106MORC30] Sonnleitner E, Bläsi U. 2014 Regulation of Hfq by the RNA CrcZ in *Pseudomonas aeruginosa* carbon catabolite repression. PLoS Genet 10: e1004440 10.1371/journal.pgen.1004440 24945892 PMC4063720

[RNA068106MORC31] Storz G, Opdyke JA, Zhang A. 2004 Controlling mRNA stability and translation with small, noncoding RNAs. Curr Opin Microbiol 7: 140–144. 10.1016/j.mib.2004.02.015 15063850

[RNA068106MORC32] Sun X, Zhulin I, Wartell RM. 2002 Predicted structure and phyletic distribution of the RNA-binding protein Hfq. Nucleic Acids Res 30: 3662–3671. 10.1093/nar/gkf508 12202750 PMC137430

[RNA068106MORC33] Tree JJ, Granneman S, McAteer SP, Tollervey D, Gally DL. 2014 Identification of bacteriophage-encoded anti-sRNAs in pathogenic *Escherichia coli*. Mol Cell 55: 199–213. 10.1016/j.molcel.2014.05.006 24910100 PMC4104026

[RNA068106MORC34] Tsui HC, Leung HC, Winkler ME. 1994 Characterization of broadly pleiotropic phenotypes caused by an *hfq* insertion mutation in *Escherichia coli* K-12. Mol Microbiol 13: 35–49. 10.1111/j.1365-2958.1994.tb00400.x 7984093

[RNA068106MORC35] Tsui HC, Feng G, Winkler ME. 1996 Transcription of the *mutL* repair, *miaA* tRNA modification, *hfq* pleiotropic regulator, and *hflA* region protease genes of *Escherichia coli* K-12 from clustered Eσ^32^-specific promoters during heat shock. J Bacteriol 178: 5719–5731. 10.1128/jb.178.19.5719-5731.1996 8824618 PMC178412

[RNA068106MORC36] Tsui HC, Feng G, Winkler ME. 1997 Negative regulation of *mutS* and *mutH* repair gene expression by the Hfq and RpoS global regulators of *Escherichia coli* K-12. J Bacteriol 179: 7476–7487. 10.1128/jb.179.23.7476-7487.1997 9393714 PMC179700

[RNA068106MORC37] Updegrove TB, Zhang A, Storz G. 2016 Hfq: the flexible RNA matchmaker. Curr Opin Microbiol 30: 133–138. 10.1016/j.mib.2016.02.003 26907610 PMC4821791

[RNA068106MORC38] Vecerek B, Moll I, Bläsi U. 2005 Translational autocontrol of the *Escherichia coli hfq* RNA chaperone gene. RNA 11: 976–984. 10.1261/rna.2360205 15872186 PMC1370782

[RNA068106MORC39] Vogel J, Luisi BF. 2011 Hfq and its constellation of RNA. Nat Rev Microbiol 9: 578–589. 10.1038/nrmicro2615 21760622 PMC4615618

[RNA068106MORC40] Vogel J, Bartels V, Tang TH, Churakov G, Slagter-Jäger JG, Hüttenhofer A, Wagner EG. 2003 RNomics in *Escherichia coli* detects new sRNA species and indicates parallel transcriptional output in bacteria. Nucleic Acids Res 31: 6435–6443. 10.1093/nar/gkg867 14602901 PMC275561

[RNA068106MORC41] Vytvytska O, Jakobsen JS, Balcunaite G, Andersen JS, Baccarini M, von Gabain A. 1998 Host factor I, Hfq, binds to *Escherichia coli ompA* mRNA in a growth rate-dependent fashion and regulates its stability. Proc Natl Acad Sci 95: 14118–14123. 10.1073/pnas.95.24.14118 9826663 PMC24336

[RNA068106MORC42] Zhang A, Wassarman KM, Rosenow C, Tjaden BC, Storz G, Gottesman S. 2003 Global analysis of small RNA and mRNA targets of Hfq. Mol Microbiol 50: 1111–1124. 10.1046/j.1365-2958.2003.03734.x 14622403

[RNA068106MORC43] Zhang A, Schu DJ, Tjaden BC, Storz G, Gottesman S. 2013 Mutations in interaction surfaces differentially impact *E. coli* Hfq association with small RNAs and their mRNA targets. J Mol Biol 425: 3678–3697. 10.1016/j.jmb.2013.01.006 23318956 PMC3640674

